# Synergistic Interface Layer Optimization and Surface Passivation with Fluorocarbon Molecules toward Efficient and Stable Inverted Planar Perovskite Solar Cells

**DOI:** 10.34133/2021/9836752

**Published:** 2021-06-28

**Authors:** Long Zhou, Jie Su, Zhenhua Lin, Xing Guo, Jing Ma, Tao Li, Jincheng Zhang, Jingjing Chang, Yue Hao

**Affiliations:** ^1^State Key Discipline Laboratory of Wide Band Gap Semiconductor Technology, Shaanxi Joint Key Laboratory of Graphene, School of Microelectronics, Xidian University, 2 South Taibai Road, Xi'an 710071, China; ^2^Centre for Spintronics and Quantum System, State Key Laboratory for Mechanical Behavior of Materials, School of Materials Science and Engineering, Xi'an Jiaotong University, Xi'an, Shaanxi 710049, China; ^3^Advanced Interdisciplinary Research Center for Flexible Electronics, Xidian University, 2 South Taibai Road, Xi'an 710071, China

## Abstract

Large-size organic halide passivation has been considered an efficient approach to enhance the perovskite solar cell (PSC) efficiency and stability. Herein, a facile posttreatment strategy was demonstrated, wherein trifluoromethyl-phenethylamine hydrobromide (CF_3_-PEABr) is firstly used to passivate the perovskite film surface. The CF_3_-PEABr surface posttreatment could coordinate with halide dangling bonds that exist at the perovskite crystal surface. Moreover, the surface treatment with CF_3_-PEABr could efficiently passivate the defects in the perovskite film and suppress the nonradiative carrier recombination. As a result, a high efficiency of 21.3% is obtained, and an increment of 80 mV in *V*_oc_ (a large *V*_oc_ of 1.15 V, with a 0.42 V voltage deficit) occurs, compared to the control device. To relieve the hydrophobic nature properties of the -CF_3_ functional group and the dewetting problem of PCBM layer deposition, a surfactant Triton X-100 is used to modify the PCBM layer. Furthermore, the devices with CF_3_-PEABr posttreatment exhibit better operational, thermal (85°C), and long storage stabilities without any encapsulation.

## 1. Introduction

The organic-inorganic hybrid perovskite has received increasing attention owing to its unique and remarkable optoelectronic properties [1–8]. The certified power conversion efficiency (PCE) of single-junction solar cells based on perovskite thin films has reached 25.5% until now [9]. Thus, the perovskite solar cell (PSC) has been heralded as a new-generation photovoltaic technology owing to its low-cost and solution-processed fabrication [10–13]. Despite such brilliant outcomes, both device efficiency and stability are found to be dramatically degenerated by the defects existing in amorphous regions, film surfaces, and grain boundaries [14–17]. To reduce defects in perovskite films and further improve the device efficiency and stability, miscellaneous strategies were exploited, such as composition engineering, additive engineering, interface layer engineering, and surface passivation engineering [18–22]. Meanwhile, massive energy loss (*E*_loss_) observed in perovskite solar cells could impair the ultimate efficiency, which suggests that the nonradiative recombination is induced by the trap-state density and defects in perovskite films [23, 24].

Up to now, long-chain cations or large-radius organic cations are widely used in surface passivation engineering, which could further enhance the device open-circuit voltage (*V*_oc_) and fill factor (FF) by suppressing the defects in the perovskite film [25–29]. Recently, Zheng et al. demonstrated a trace amount of surface-anchoring alkylamine ligands to modify the interface and grain, which could enhance the charge carrier mobility and reduce the trap-state density of perovskite films [30]. They achieved a certified stabilized PCE of 22.3% with an excellent *V*_oc_ of 1.17 V and remarkable operating stability with over 1000 h under continuous illumination. Yang et al. designed a new passivation molecule, wherein D-4-tert-butylphenylalanine was used to passivate perovskite defects by combining all effective passivation groups [31]. They reported a remarkable small *V*_oc_ deficit of 0.34 V and a high efficiency of 21.6% with a large *V*_oc_ of 1.23 V. Furthermore, Gharibzadeh et al. reported a significant improvement via mixed 2D/3D perovskites to obtain an efficiency of 19.4% and a large *V*_oc_ of 1.31 V [32]. Additionally, Jiang's research group used a large organic salt phenethylammonium iodide (PEAI) to modify the perovskite film surface for defect passivation, and a certified efficiency of 23.32% with a large *V*_oc_ of 1.18 V was obtained, which approximated to the Shockley-Queisser limit [14]. Zhou et al. firstly demonstrated that by introducing 2-(4-fluorophenyl)ethyl ammonium iodide and a fluorinated aromatic cation to form 2D/3D perovskites as the absorbing layer, the photovoltaic devices achieved a high *V*_oc_ of 1.12 V, leading to a stabilized efficiency of 20.54%, and the device maintained 99% of the initial efficiency after 864 h [33]. Thereafter, Zhou et al. investigated the fluorination position (ortho-, meso-, and para-) effect on the aromatic moiety and obtained a PCE of 20.1% with a remarkable *V*_oc_ of 1.21 V [34]. Recently, Zhu et al. used fluorinated BAI to treat the perovskite film and introduced 4-(trifluoromethyl)pyridine (TFP) as an additive to increase the HTL's hydrophobicity, which could suppress the nonradiative charge carrier recombination and enhance the PSC's resistance to moisture and device stability [35]. As viewed by these results, the search for alternative hydrophobic large cations for perovskite passivation to achieve high performance is still a challenge. As a result, it is highly significant to achieve larger *V*_oc_ and high efficiency for further commercial applications of the optoelectronic device.

Herein, we demonstrated a novel approach to modify the perovskite surface via depositing phenethylamine hydrobromide with the trifluoromethyl functional group (-CF_3_) and ammonium group to suppress the trap-state density and improve the photovoltaic performance and moisture stability. A large organic salt containing positively charged cations (CF_3_-PEA^+^) could coordinate with halide dangling bonds that exist at the crystal surface and passivate defects arising from vacancies of halide ions or organic cations on the perovskite film [36, 37]. Meanwhile, we introduced a surfactant (Triton X-100) to modify the PCBM layer, which could provide potential interaction with the perovskite surface due to the hydrophilic polyethylene oxide chain and self-assemble on the surface of the PCBM layer with the hydrophobic group. As a result, the device with the synergistic effect of CF_3_-PEABr modification and surfactant Triton X-100 demonstrates a high PCE of 21.3% with a large *V*_oc_ of 1.15 V and an excellent FF of 81.4%. Moreover, the CF_3_-PEABr-modified device shows better stability than the conventional device.

## 2. Results and Discussion

**Figure 1:**
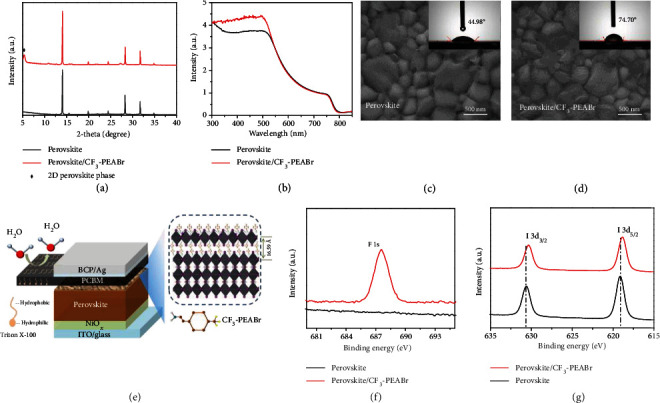
Perovskite material characterization and passivation schematic. (a) XRD spectra of conventional and CF_3_-PEABr-treated perovskite films. (b) Absorption spectra of conventional and CF_3_-PEABr-treated perovskite films. (c, d) SEM images of conventional and CF_3_-PEABr-treated perovskite films. Inset shows the water contact angle images. (e) Schematic model of the perovskite solar cell with CF_3_-PEABr passivation. (f, g) XPS curves of perovskite films with or without CF_3_-PEABr passivation.

The perovskite film MA_1-*y*_FA*_y_*PbI_3-*x*_Cl*_x_* was deposited using the two-step spin coating method, which is reported in our previous works [7, 11]. After perovskite film annealing, the CF_3_-PEABr/IPA solution (2 mg/mL in IPA) was used to treat the perovskite film surface further, generating a 2D/3D perovskite heterostructure [7]. We firstly investigated the XRD spectra of perovskite films with and without CF_3_-PEABr treatment, as shown in Figure 1(a). Both films exhibit obviously intense diffraction peaks located at 14.1°, 28.4°, and 31.8° which correspond to the (110), (220), and (310) typical crystal planes of the tetragonal phase. It is obvious that the peak located at 5.4° appears in the perovskite film with CF_3_-PEABr passivation, which means the formation of low-dimensional perovskites. To further clarify that low-dimensional perovskite structure, we measured the XRD pattern of 2D perovskite (CF_3_-PEA)_2_PbI_2_Br_2_ prepared by CF_3_-PEABr and PbI_2_. The low-angle diffraction peak of mixed 2D/3D perovskite measured is consistent with the peak of 2D perovskite (CF_3_-PEA)_2_PbI_2_Br_2_ (Figure S1). Meanwhile, the *d*-space of low-dimensional perovskites was calculated as 16.38 Å using Bragg's law which is also in good agreement with the 2D perovskite (CF_3_-PEA)_2_PbI_2_Br_2_'s value (16.59 Å) [13]. Furthermore, low-dimensional perovskites could be confirmed by the PL spectrum that a small peak corresponding to low-dimensional perovskites appears at around 525 nm (Figure S2). Moreover, the peak intensity of perovskite films with CF_3_-PEABr treatment is slightly higher than that of conventional films, indicating higher film crystallinity with surface passivation. Meanwhile, a slightly narrowed full width at half maximum (FWHM) can be observed for perovskite films with CF_3_-PEABr treatment, which means less defect density, higher crystallinity, and better film quality, as shown in Figure 1(a) and Figure S2. Figure 1(b) depicts the UV-vis absorption spectra of all perovskite films. The optical band edge of conventional perovskite films is located at 790 nm, consisting of a bandgap of 1.57 eV. There is a blueshift behavior for the optical band edge of perovskite films with CF_3_-PEABr surface passivation, indicating a larger optical bandgap for the top perovskite thin film (Figure S3). This result is in good agreement with other large cations reported in previous works [29]. Notably, the perovskite film with CF_3_-PEABr treatment exhibits higher absorption intensity, which is profited from its high film quality and surface passivation. Furthermore, the surface morphology of perovskite films was investigated via scanning electron microscopy (SEM) measurement in Figures 1(c) and 1(d). Uniform and dense morphologies are observed for both films. It is obvious that stripy grains are observed in the perovskite film with CF_3_-PEABr passivation, which means that the large organic cations CF_3_-PEA^+^ caused layer structure formation. Notably, CF_3_-PEABr has an alkyl hydrophobic chain on account of its -CF_3_ functional group. We further investigated the water contact angles of perovskite films with or without CF_3_-PEABr treatment. By contrast, the perovskite film with CF_3_-PEABr displays a much larger water contact angle (74.70°) than the conventional film (44.98°). The result indicates that the CF_3_-PEABr assembles on the perovskite film surface, which dramatically increases the hydrophobicity of the film. This hydrophobicity could not only potentially increase the film stability but also generate the dewetting problem of PCBM layer deposition. Furthermore, we investigated the roughness of the perovskite film surface via atomic force microscopy (AFM) measurement, as shown in Figure S4. The perovskite film with CF_3_-PEABr passivation exhibits smaller roughness (11.8 nm) than the conventional film (15.4 nm), which is advantageous for upper layer deposition and interface quality. As shown in Figure 1(e), the unavoidable loss of halide ions or organic cations on the surface of conventional perovskite films leads to defects (including halide interstitial, halide vacancy, and Pb vacancy) because of the small formation energy [38]. In principle, CF_3_-PEABr contains an ammonium group and a trifluoromethyl group. The ammonium group could form hydrogen bonding with halide ions grown on the surface of perovskites due to stronger electrostatic interactions. As a result, the CF_3_-PEABr can suppress the formation of halide dangling bonds, vacancies, and defects located at grain boundaries [39, 40]. On the other hand, the ultrathin low-dimensional perovskite formed on the 3D perovskite surface due to the induced CF_3_-PEA^+^ ions and the hydrophobic trifluoromethyl functional group -CF_3_ exists at the terminal of the crystal, acting as the barrier to prevent the H_2_O from ambient air into perovskite films in high-humidity environments. Similar results have been verified by X-ray photoelectron spectroscopy (XPS), as shown in Figures 1(f) and 1(g). It is obvious that the emerged F 1s peaks (at 687.6 eV) and C-F bond are mainly attributed to the incorporation of CF_3_-PEA^+^ cations for the film with surface passivation, indicating the presence of CF_3_-PEABr on the film surface. Furthermore, the smaller binding energy of the I peak is discovered after CF_3_-PEABr passivation, which mainly results from the strong positively charged NH_3_^+^ end of the CF_3_-PEABr molecule. The NH_3_^+^ end of the CF_3_-PEABr molecule could grow on the surface of perovskite films and form a hydrogen bond with halide ions, which weakens the interaction between Pb and I [36]. Due to the electron-donating nature of the Lewis base, the Pb and I peaks of the film with CF_3_-PEABr treatment shifted toward lower binding energy, which further verifies the interaction between surface defects such as the uncoordinated Pb^2+^ or halide dangling bond and CF_3_-PEABr molecule. Meanwhile, the C=O peak located at 288.1 eV is observed and mainly induced by the decomposition under ambient conditions during measurement (Figure S6), while the intensity of the C=O peak obviously decreases after CF_3_-PEABr passivation, corroborating the better moisture stability due to the hydrophobic trifluoromethyl functional group. Moreover, the modification of CF_3_-PEABr is further confirmed by the N 1s region in X-ray photoelectron spectroscopy (XPS). The N 1s spectrum reveals that the typical bond of the C-NH_2_ group is induced by FA^+^ cations or CF_3_-PEA^+^ cations and the bond of the C=NH_2_^+^ group is induced by FA^+^ cations in the perovskite film. Compared with the control perovskite film, the perovskite/CF_3_-PEABr film exhibits a higher ratio of the C-NH_2_ group which is mainly attributed to the introduction of CF_3_-PEABr. Furthermore, it is obvious that the peaks of the perovskite/CF_3_-PEABr film shift toward lower binding energy, which further suggests that CF_3_-PEABr has a strong molecular interaction with the perovskite surface. Fourier transform infrared spectroscopy (FTIR) measurements are used to confirm the possible chemical interaction in perovskite films. The strengthening and broadened profile of the N-H bending vibration band (1550−1600 cm^−1^) could further demonstrate the molecular interaction in the perovskite/CF_3_-PEABr film compared with the control film (Figure S5) [13, 41, 42].

**Figure 2:**
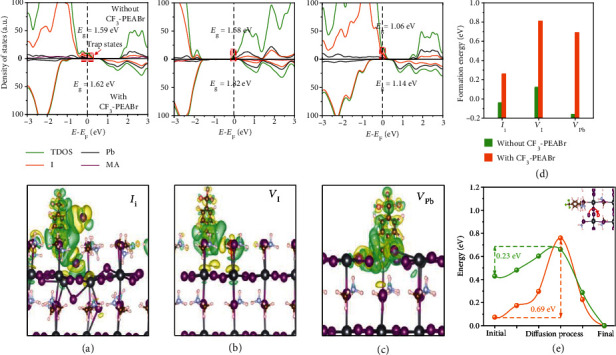
DFT calculation for CF_3_-PEABr passivation. (a–c) Density of states and charge density difference of the CF_3_-PEABr-passivated MAI surface with (a) *I*_i_ and (b) *V*_I_ and the (c) PbI surface with *V*_Pb_. (d) Formation energies of *I*_i_, *V*_I_, and *V*_Pb_ at the perovskite surface. (e) The diffusion energy curves of iodide ion in the MAI surface with and without CF_3_-PEABr passivation, where the migration energy is marked by the arrows. The inserts are the diffusion paths in the perovskite.

To reveal the passivation mechanism of CF_3_-PEABr, we calculated the density of states (DOS) and charge transfer of MAPbI_3_ absorbed with CF_3_-PEABr by density functional theory (DFT). Both the I interstitial (*I*_i_) and I vacancy (*V*_I_) at the MAI-terminated surface and the Pb vacancy (*V*_Pb_) at the PbI-terminated surface were considered. As shown in Figure 2(a), the *I*_i_ introduces a defect state in the bandgap (about 1.59 eV) and serves as a hole trap. The CF_3_-PEABr could supply electrons to the perovskite and passivate these hole trap states, suggesting reduced nonradiative recombination for CF_3_-PEABr-passivated perovskite. Meanwhile, such electron transfer could tune the position of the Fermi level related to the band edges of perovskite and then improve the band offset. As a result, the bandgap of perovskite is slightly enlarged by the CF_3_-PEABr, which is consistent with the above experimental analysis. A similar electron transfer and enlarged bandgap are also observed for CF_3_-PEABr that passivated the *V*_I_ and *V*_Pb_. Meanwhile, no defect states are formed in the bandgap upon absorbing the CF_3_-PEABr, as exhibited in Figures 2(b) and 2(c). In addition, it could be observed from Figure 2(d) that the formation energy of these defects increases after incorporation of CF_3_-PEABr since the Br anion could occupy the *V*_I_, and the H of the ammonium group could bond with the iodine dangling bond (as displayed in Figures 2(a)–2(c)). These bonds significantly reduce the initial energy of the I ion migration process and then enlarge the migration energy of I ion and suppress I ion migration, as displayed in Figure 2(e). As a result, the CF_3_-PEABr preferentially bounds to the perovskite surface and stabilizes the perovskite surface structure.

**Figure 3:**
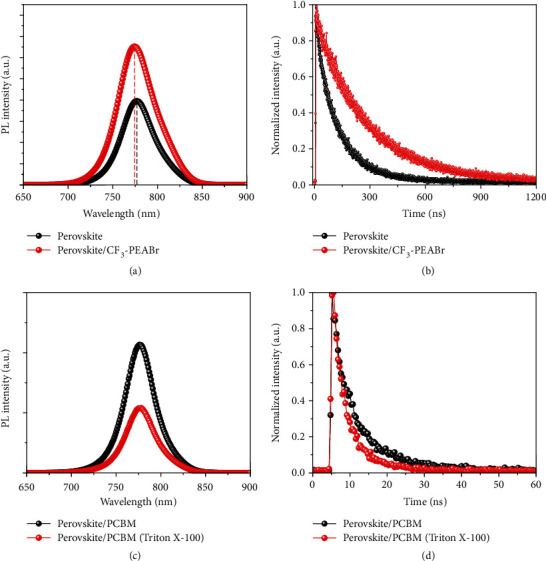
Carrier dynamics for perovskite films. (a) PL and (b) TRPL spectra of perovskite films with or without CF_3_-PEABr treatment. (c) PL and (d) TRPL spectra of perovskite films with PCBM or PCBM (Triton X-100) layers.

To investigate the carrier dynamics in perovskite films with the effect of CF_3_-PEABr passivation, we carried out the steady-state photoluminescence (PL) measurement and time-resolved PL (TRPL) decay measurements, as shown in Figures 3(a) and 3(b). The carrier lifetime can be extracted with a double exponential decay model [42], as listed in Table S1. After CF_3_-PEABr surface passivation, the increased peak intensity and blueshift behavior are observed, indicating efficient surface passivation and lower trap-state density in perovskite films. Meanwhile, the perovskite film with CF_3_-PEABr passivation possesses a longer carrier lifetime (269.1 ns) than the conventional film (127.4 ns), indicating that nonradiative carrier recombination is effectively suppressed. In addition, we have checked the effect of surface passivation on the band alignment via ultraviolet photoelectron spectroscopy (UPS) measurement, as shown in Figure S7. We found that the perovskites with CF_3_-PEABr passivation showed a smaller work function value and better band alignment between the perovskite and the PCBM layer, which indicated an efficient charge transfer process between the perovskite and the PCBM layer. Furthermore, work functions were further checked with surface potential via Kelvin probe force microscopy (KPFM), as shown in Figure S8. A higher surface potential for the perovskite film with CF_3_-PEABr treatment was discovered, indicating the decreased work function with the CF_3_-PEABr modification. The apparent decrease in work functions of perovskites with CF_3_-PEABr modification could form a band bending between the perovskite and the PCBM layer, which could reconfigure the interfacial energy band structure, leading to enhanced built-in potential and charge collection.

The poor coverage of the PCBM layer on the hydrophobic perovskite film is another challenge preventing excellent device performance. As discussed above, the perovskite film with CF_3_-PEABr treatment has evident hydrophobic properties owing to the -CF_3_ functional group, arousing the problem that the PCBM layer cannot be successfully spin-coated on the perovskite film. For the CF_3_-PEABr surface passivation, the devices with a pure PCBM layer exhibited a terrible PCE (Figure S9) due to the poor PCBM coverage caused by the hydrophobic surface of the perovskite film. Thus, it is necessary to use a surfactant to relieve the nonwetting problem of the perovskite film. Triton X-100 is one of the nonionic surfactants and is widely used in biochemical and industrial processes due to its amphiphilic structure. The Triton X-100 molecule includes a hydrophilic polyethylene oxide chain and an aromatic hydrocarbon group, as shown in Figure S10. Thus, here, we introduced a surfactant (Triton X-100) with a hydrophilic polyethylene oxide chain to modify the PCBM layer. And Triton X-100 exhibits a terminal hydroxyl group, which could provide potential interaction such as hydrogen bonding or electrostatic interaction with the CF_3_ of the perovskite film surface, which can promote the successful PCBM deposition on the perovskite film with CF_3_-PEABr passivation. Furthermore, we investigated the carrier transport kinetics between the perovskite and the PCBM layer, as shown in Figures 3(c) and 3(d). By contrast, obvious PL quenching behavior is observed for the perovskite/PCBM (Triton X-100), which indicates the efficient carrier transport from the perovskite to the PCBM layer. Additionally, the lifetime of the sample with PCBM (Triton X-100) (4.30 ns) is smaller than that of the sample with PCBM (7.24 ns), which validates that the surfactant (Triton X-100) could improve the interfacial contact between PCBM and CF_3_-PEABr, which enhances the electron extraction and transport from the perovskite to the PCBM layer (Table S2).

**Figure 4:**
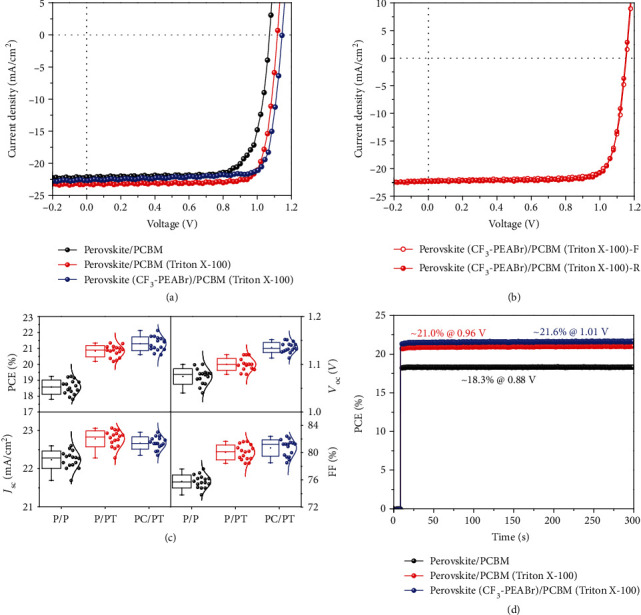
Device performance of PSCs for different conditions. (a) *J*-*V* curves of perovskite devices with different conditions. (b) Hysteresis behaviors of perovskite devices based on perovskite (CF_3_-PEABr)/PCBM (Triton X-100). (c) Distribution of device performance with different conditions. P/P: perovskite/PCBM; P/PT: perovskite/PCBM (Triton X-100); PC/PT: perovskite (CF_3_-PEABr)/PCBM (Triton X-100). (d) Steady-state output of current density and PCE at the maximum power point.

In order to provide insight into the effect of the CF_3_-PEABr surface passivation, the inverted planar p-i-n-type PSC devices were fabricated. The architecture of the solar cell based on the NiO*_x_* hole transport layer is presented in Figure 1(e).  Figure 4(a) shows the current density-voltage (*J*-*V*) curves of devices, and Table 1 lists the detailed parameters of device performance. The control device exhibits an average PCE of 18.5%, a *V*_oc_ of 1.07 V, a *J*_sc_ of 22.2 mA/cm^2^, and an FF of 76.5%. In contrast, the device with synergistic passivation of CF_3_-PEABr treatment and surfactant (Triton X-100) modification shows an increased PCE of 21.3% with a large *V*_oc_ of 1.15 V, a *J*_sc_ of 22.7 mA/cm^2^, and an FF of 81.4%. The champion device exhibits a high PCE of 21.9%. The slightly improved current density mainly results from the ultrathin low-dimensional perovskite formed on the 3D perovskite surface, and excess amounts of large cations absorbed on the perovskite crystal surface could physically limit carrier transport and lead to the decreased photocurrent [43], which has been verified in the above results. The device has a significant improvement in PCE than the control device due to the great enhancement in *V*_oc_ and FF, which may result from the higher-quality perovskite film, lower trap density, and better band alignment (Figure S7). In comparison, the device with PEABr surface passivation also fabricated and exhibited a PCE of 20.6% with a *V*_oc_ of 1.12 V, but this *V*_oc_ is still smaller than that of the device with CF_3_-PEABr passivation because of the electron-withdrawing -CF_3_ group introduction (Figure S11). CF_3_-PEABr could provide hydrogen bonding between H of the ammonium group and halide ions or electrostatic interactions which can be credited to the electron-withdrawing F atom. As a result, it can avoid the formation of halide dangling bonds and vacancy-related defects and efficiently improve film quality. In order to check the effect of surfactant Triton X-100, we fabricated the device with a modified PCBM (Triton X-100) layer based on the conventional perovskite film. Unexpectedly, it showed a significantly improved PCE of 21.0% as a consequence of the increased *J*_sc_ of 23.3 mA/cm^2^, *V*_oc_ of 1.11 V, and FF of 80.7%, compared with the conventional device. It can be concluded that the *J*_sc_ improvement is mainly caused by Triton X-100 modification, while the *V*_oc_ improvement is mainly caused by CF_3_-PEABr surface passivation, and the synergistic effect could be achieved to obtain the best device performance. All samples with modification of CF_3_-PEABr or Triton X-100 have been carefully optimized with different concentrations to achieve the best performance (Figures S12 and S13). Additionally, negligible hysteresis behavior appeared in both the forward and reverse scan directions for all samples, as shown in Figure 4(b) and Figure S14. In addition, the stronger electrostatic interactions and hydrogen bonding coordination via CF_3_-PEABr posttreatment upon the surface can block the accumulation of charge at the interface. Meanwhile, Figure 4(c) shows the device performance distribution of PSCs under different conditions, indicating good reliability and uniformity for the device fabrication. To further verify device *J*_sc_, the external quantum efficiency (EQE) spectra and the integrated *J*_sc_ are depicted in Figure S15. The device with a modified PCBM (Triton X-100) layer based on the conventional perovskite film and CF_3_-PEABr passivation has a larger integrated *J*_sc_ value (22.8 mA/cm^2^ and 22.3 mA/cm^2^), which is much higher than that of the conventional device (21.9 mA/cm^2^). All of these results are in good agreement with the results extracted from measured *J*-*V* curves. Moreover, we have measured the steady-state output of PCE values at the maximum power point (Figure 4(d)). The devices exhibit a steady-state PCE of 18.3%, 21.0%, and 21.6% under different conditions, respectively. All of them show the excellent reliability of the *J*-*V* scans and the stability of device performance. Notably, the photocurrent is stabilized within seconds when the light is turned on, which supports the hysteresis-free behavior of the devices.

**Figure 5:**
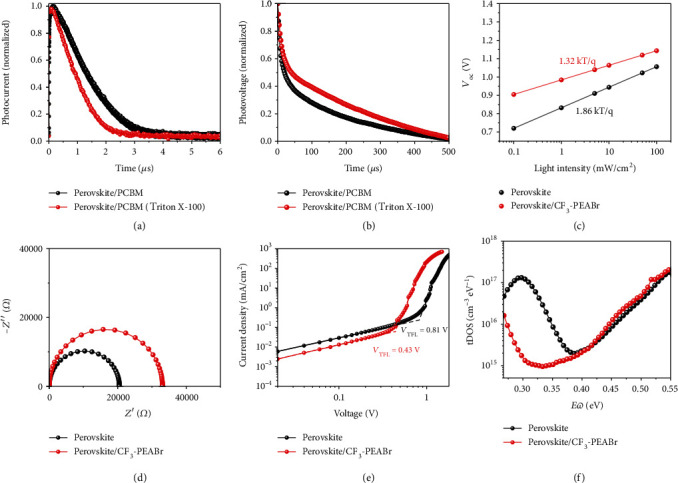
Device-level characterization for different conditions. (a) Transient photocurrent and (b) transient photovoltage measurements of the conventional device and the device with Triton X-100. (c) *V*_oc_ as a function of light intensity. (d) EIS of the devices with or without CF_3_-PEABr treatment. (e) *I*-*V* curves for electron-only devices. (f) tDOS curves of perovskite solar cells with or without CF_3_-PEABr passivation.

In addition, we provide insight into the carrier transport and recombination mechanism via the transient photocurrent (TPC) and transient photovoltage (TPV) measurements. Both of them are used to explain the larger *J*_sc_ caused by the effect of surfactant Triton X-100, as shown in Figures 5(a) and 5(b). The photocurrent decay lifetime is related to the charge extraction and transport processes, and the photovoltage decay lifetime mainly indicates the charge recombination processes. Noteworthily, the device with surfactant Triton X-100 exhibits faster decay behavior (0.81 *μ*s) than the conventional device (1.32 *μ*s), indicating more efficient charge extraction and transport near the perovskite surface, which conformed well with EQE results.  Figure 5(b) reveals that the photovoltage lifetime of the device with surfactant Triton X-100 (437 *μ*s) is larger than that of the conventional device (228 *μ*s), indicating the suppressed charge recombination near the perovskite surface. The better carrier transport and suppressed charge recombination are benefited from the interaction between Triton X-100 and perovskite. All of these results are in good agreement with the increased *J*_sc_ for Triton X-100-modified PCBM-based devices.

In order to further check the effect of CF_3_-PEABr passivation and surfactant Triton X-100 on carrier extraction and transport mechanisms, the relationship of *V*_oc_ as a function of light intensity was investigated, as shown in Figure 5(c). The *V*_oc_ is measured with respect to the light intensity at various light intensities, from 100 to 0.1 mW/cm^2^. There is a linear relationship with a slope of *K*_B_*T*/*q* between *V*_oc_ and light intensity on a semilogarithmic scale, where *K*_B_ is the Boltzmann constant, *T* is the absolute temperature, and *q* is the elementary charge, respectively. The slope extracted from the curves of the device with CF_3_-PEABr passivation (1.32 *K*_B_*T*/*q*) is much smaller than that of the conventional device (1.86 *K*_B_*T*/*q*), which suggests that CF_3_-PEABr surface passivation results in the minimized energy loss and suppresses carrier recombination with strong coordination. To further confirm the charge transport and recombination process, the electrical impedance spectroscopy (EIS) measurement was carried out to extract the recombination resistance (*R*_rec_) and series resistance (*R*_s_). The devices are measured in the dark condition with an applied voltage of 1 V, and the corresponding Nyquist is plotted in Figure 5(d). Meanwhile, the fitting parameters extracted with the equivalent circuit are listed in Table S3. The device with CF_3_-PEABr passivation exhibits larger *R*_rec_ than the conventional device, indicating the suppressed carrier recombination. This result is well consistent with the *R*_sh_ results extracted from *J*-*V* curves and the larger *V*_oc_ of the device with CF_3_-PEABr passivation. Furthermore, the capacitance-voltage (*C*-*V*) characteristics of devices were operated to check the built-in potential (*V*_bi_), which could be extracted with the Mott-Schottky method [44], as shown in Figure S16. The measurable enhanced *V*_bi_ (1.13 V) compared with that of the conventional device (0.95 V) means a promoted driving force for photogenerated carrier separation as well as an efficiently suppressed electron-hole recombination. In order to further verify the trap-state density of perovskite films, electron-only devices (ITO/SnO_2_/perovskite/PCBM/Ag) were fabricated. Thus, the trap-filled limit voltage (*V*_TFL_) is extracted from *I*-*V* curves and the trap-state density (*n*_trap_) is determined from the trap-filled limit voltage according to the equation *V*_TFL_ = *en*_trap_*L*^2^/2*ε*_0_*ε*, where *e* is the electron charge, *L* is the thickness of the electron-only device, *ε* is the relative dielectric constant, and *ε*_0_ is the vacuum permittivity [45, 46], as shown in Figure 5(e). It is noticed that a smaller *V*_TFL_ value (0.43 V) could be obtained for the perovskite film with CF_3_-PEABr passivation compared with the conventional device (0.81 V), which indicates that the perovskite film with large cation passivation owns significantly reduced electron trap density (0.86 × 10^15^ cm^−3^) than the conventional film (1.62 × 10^15^ cm^−3^). Furthermore, a reduced trap density in devices with CF_3_-PEABr passivation was measured by thermal admittance spectroscopy, as shown in Figure 5(f). The device with CF_3_-PEABr passivation exhibited lower trap density of states (tDOS) in the shallow trap depth region (0.25 to 0.35 eV), which is attributed to the passivation of surface defects. A lower shallow trap density means that the defects at grain boundaries were efficiently passivated via the effect of CF_3_-PEABr for perovskites. The lower trap density should be associated with the coordination between CF_3_-PEABr and halide dangling bonding located at the perovskite surface. All of these results are well consistent with the material characterization.

**Figure 6:**
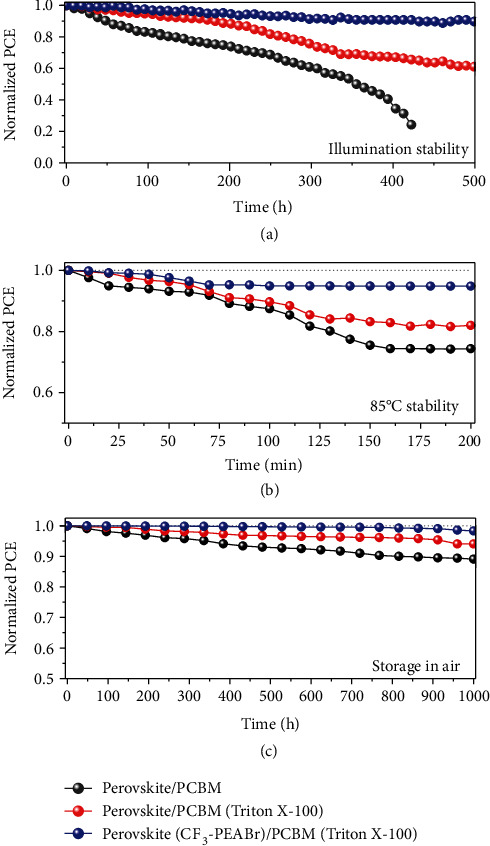
Film and device stability for different conditions. (a) Operational stability of the device with epoxy encapsulation. (b) Continuous heating at 85°C in ambient air. (c) Moisture stability under RH ~ 35% condition.

To further investigate the effect of CF_3_-PEABr passivation on device stability, we measured the illumination stability, 85°C thermal stability, and long storage stability of the devices. Firstly, we measured the light soaking stability of the perovskite film without encapsulation under 1.7 sunlight. It is obvious that the film with CF_3_-PEABr passivation shows much greater light stability than the control film. The passivated perovskite film still remains black in color while the control film has turned yellow after 24 h of illumination, as shown in Figure S17. Moreover, Figure 6(a) shows the illumination stability of the device that was measured for 500 h under one sunlight. The device was encapsulated by epoxy and glass. We demonstrate that the device with CF_3_-PEABr passivation shows better operational light stability than the conventional device. After continuous light illumination for 500 h, the PCE of the device with CF_3_-PEABr passivation could retain 90% of the initial efficiency which is much higher than that of the conventional device. Fortuitously, the device with a modified PCBM (Triton X-100) layer also retains 60% of its initial PCE. Moreover, the thermal stability of devices with different conditions was measured under 85°C continuous heating, as shown in Figure 6(b). It is found that the device based on the perovskite with CF_3_-PEABr passivation still retains 94% of the initial PCE after 200 min without encapsulation, while the device without aromatic cation passivation exhibits poor stability (74% of the initial PCE for the conventional device and 82% of the initial PCE for the device with a modified PCBM (Triton X-100) layer). In addition, we measured the long-term storage stability in ambient air with RH ~ 35% for 1000 h, as shown in Figure 6(c). As expected, the device with CF_3_-PEABr passivation exhibits better PCE stability, retaining 98% of the initial PCE. Meanwhile, the device with a modified PCBM (Triton X-100) layer also retains 94% of the initial PCE, larger than that of the conventional device (89%). Absolutely, the improved stability of target devices is ascribed to the lower trap-assisted states and hydrophobic surface of perovskites due to the hydrophobic nature of CF_3_-PEABr. The hydrophobic trifluoromethyl functional group -CF_3_-PEA^+^ that existed at the terminal of the crystal acts as the dynamic barrier to prevent the H_2_O or O_2_ from ambient air into perovskite films in high-humidity environments.

## 3. Conclusion

In summary, we demonstrate an efficient strategy of surface passivation, wherein CF_3_-PEABr large organic cations with the electron-withdrawing trifluoromethyl functional group could form coordination with halide dangling bonds at the perovskite crystal surface and reduce trap-assisted states that existed in the perovskite film, leading to efficiently increased perovskite film quality and suppressed nonradiative carrier recombination. Meanwhile, a surfactant Triton X-100 is adopted to solve the hydrophobic nature properties of the -CF_3_ functional group and provide potential interaction between the PCBM layer and the perovskite to enhance charge transfer near the perovskite surface. As a result, the PSCs with CF_3_-PEABr surface passivation show significantly enhanced efficiency and better stability, wherein a higher PCE of 21.3% and a larger *V*_oc_ of 1.15 V are obtained. The lower energy loss (0.42 eV) is related to the reduced defects in perovskite films and suppressed nonradiative carrier recombination. Meanwhile, the device with CF_3_-PEABr surface passivation shows better light soaking, 85°C thermal, and long-term storage stabilities without any encapsulation after 1000 h. Our results provide a facile way to enhance the performance and the stability of perovskite solar cells.

## Figures and Tables

**Table 1:** Average device performance parameters for perovskite solar cells with different modifications. The average PCE data were calculated from at least 36 devices.

Condition	*V*_oc_ (V)	*J*_sc_ (mA/cm^2^)	FF (%)	PCE (%) (best device)	*R*_s_ (Ω cm^2^)	*R*_sh_ (kΩ cm^2^)
Perovskite/PCBM	1.07 ± 0.04	22.2 ± 0.6	76.5 ± 3.2	18.5 (19.6)	3.13	1.51
Perovskite/PCBM (Triton X-100)	1.11 ± 0.02	23.3 ± 0.5	80.7 ± 2.5	21.0 (21.5)	2.68	3.45
Perovskite (CF_3_-PEABr)/PCBM (Triton X-100)	1.15 ± 0.02	22.7 ± 0.3	81.4 ± 2.8	21.3 (21.9)	2.94	2.78

## Data Availability

Data used to support the findings of this study are included within the article and supplementary information file(s).

## Supplementary Materials

Supplementary Materials Experimental details. XRD spectra of 2D perovskites (CF_3_-PEA)_2_PbI_2_Br_2_. FWHM and PL perovskites. Tauc plot spectra of perovskite films. AFM images of perovskite films. FTIR spectra of CF_3_-PEABr powder and perovskites. XPS spectra of the perovskite films. UPS results of perovskites and band energy level scheme. KPFM images of perovskite films. Device performance for the sample with or without Triton X-100. The schematic chemical structure of Triton X-100. The performance of the device with PEABr surface passivation. Distribution of device performance for different conditions of Triton X-100. The device performance of PSCs with different concentrations of CF_3_-PEABr. Hysteresis behaviors of perovskite devices with different conditions. EQE curves of perovskite devices with different conditions. C-V curves of devices with or without CF_3_-PEABr treatment. The illumination stability of films with or without CF_3_-PEABr treatment. Cross-sectional SEM image of perovskite films. Fitting decay times of perovskite films prepared with different conditions. Fitting parameters of the EIS measurements with different conditions.
