# Supplementary material for: Synergistic Interface Layer Optimization and Surface Passivation with Fluorocarbon Molecules toward Efficient and Stable Inverted Planar Perovskite Solar Cells
Source: Research (Wash D C). 2021 Jun 28;2021:9836752. doi: 10.34133/2021/9836752 (PMC8261667; doi:10.34133/2021/9836752)
Supplement: Supplementary Materials — Experimental details. XRD spectra of 2D perovskites (CF3-PEA)2PbI2Br2. FWHM and PL perovskites. Tauc plot spectra of perovskite films. AFM images of perovskite films. FTIR spectra of CF3-PEABr powder and perovskites. XPS spectra of the perovskite films. UPS results of perovskites and band energy level scheme. KPFM images of perovskite films. Device performance for the sample with or without Triton X-100. The schematic chemical structure of Triton X-100. The performance of the device with PEABr surface passivation. Distribution of device performance for different conditions of Triton X-100. The device performance of PSCs with different concentrations of CF3-PEABr. Hysteresis behaviors of perovskite devices with different conditions. EQE curves of perovskite devices with different conditions. C-V curves of devices with or without CF3-PEABr treatment. The illumination stability of films with or without CF3-PEABr treatment. Cross-sectional SEM image of perovskite films. Fitting decay times of perovskite films prepared with different conditions. Fitting parameters of the EIS measurements with different conditions. [file 9836752.f1.docx]

Synergistic Interface Layer Optimization and Surface Passivation with Fluorocarbon Molecules toward Efficient and Stable Inverted Planar Perovskite Solar Cell

Long Zhou,^a^ Jie Su,^a^ Zhenhua Lin,^a^ Xing Guo,^a^ Jing Ma,^a^ Tao Li,^c^ Jincheng Zhang,^a,b^ Jingjing Chang,^a,b^ * and Yue Hao^a,b^

*^a^*State Key Discipline Laboratory of Wide Band Gap Semiconductor Technology, Xidian University, 2 South Taibai Road, Xi’an, 710071, China.

*^b^*Advanced Interdisciplinary Research Center for Flexible Electronics, Xidian University, 2 South Taibai Road, Xi’an, 710071, China.

^c^Centre for Spintronics and Quantum System, State Key Laboratory for Mechanical Behavior of Materials, School of Materials Science and Engineering, Xi’an Jiaotong University, Xi’an, Shaanxi 710049, China.

**Experiment section**

*Materials*: All the materials, such as methylammonium iodide (MAI), formamidinium iodide (FAI), and trifluoromethyl 2-phenylethylamine hydroiodide (CF_3_-PEABr) were purchased from Xi’an Polymer Light Technology Corp. Lead iodide (PbI_2_), lead chloride (PbCI_2_), nickel nitrate (Ni(NO_3_)_2_·6H_2_O) were brought from Sigma. N,N’-dimethylformamide (DMF), and bathocuproine (BCP) were purchased from Alfa. Phenyl-C61-butyric acid methyl ester (PCBM, 98 %) were brought from nano-c. All materials in this work were used without further purification.

*Device fabrications*: The control device fabricated process has been reported in our previous works ^1^. Additionally, for the passivation device fabrication, after perovskite film was annealed, the solution of CF_3_-PEABr (2 mg) dissolved in IPA (1 mL) was spin coated on the perovskite film and annealed at 100 ^o^C for 5 min. To avoid the effect of annealing, the control film was washed by IPA and then annealed at 100 ^o^C for 5 min

*Materials and device characterization*: All the devices were tested by using Keithley 2400 under one sun solar simulator which was calibrated with a silicon reference solar cell (NREL certified). External quantum efficiency (EQE) spectra were measured by the SCS10-X150 systems (Zolix instrument. Co. Ltd). X-ray diffraction (XRD) spectras were performed by Bruker D8 Advance XRD. The Perkin-Elmer Lambda 950 spectrophotometer was use to verify UV-vis absorption spectras. PL and TR-PL spectra were measured by the Pico Quant Fluotime 300 with a 510 nm picosecond pulsed laser. The surface morphologys of perovskite films were measured by scanning electron microscopy (SEM) (JSM-7800F). XPS and UPS measurements were performed by the Escalab 250Xi with a source of monochromatic Al-Ka (1486.6 eV). Transient photocurrent (TPC) measurements were measures with a system excited by a 532 nm (1000 Hz, 3.2 ns) pulse laser. Transient photovoltage (TPV) measurements were performed with the same system excited by a 405 nm (50 Hz, 20 ms) pulse laser. A digital oscilloscope (Tektronix, D4105) was used to record the photocurrent or photovoltage decay process with a sampling resistor of 50 Ω or 1 MΩ, respectively.

*DFT calculation:* All calculations were based on density functional theory (DFT) ^2^ as implemented in the Vienna ab initio simulation package (VASP) ^3,4^ code with projector augmented wave (PAW) method.^5,6,7^ The Perdew-Burke-Ernzerhof (PBE) functional within the generalized gradient approximation (GGA) was employed to describe the exchange-correlation interaction^8^. The migration barriers were calculated using the climbing image nudged elastic band (CI-NEB) method ^9, 10^ which is an efficient way to find saddle point configuration as implemented in VASP through the VTST tools. The plane-wave basis cutoff energy was set to be 400 eV. All structures were relaxed until the residual force on each atom less than 0.01 eV/Å. The self-consistent convergence accuracy was set at 10^-5^ eV/atom in the structural calculation, and 10^-7^ eV/atom in the CI-NEB calculation. To simplify the calculation of MA_1-y_FA_y_PbI_3-x_Cl_x_ with CF_3_-PEABr, 2 × 2 × 1 MAPbI_3_ (001) surface with five atomic-layer was employed. A vacuum region more than 15 Å in the z-direction was in conjunction with the dipole correction to avoid the fictitious interaction with its periodic images.

**Figure S1**. The XRD pattern of 2D perovskite (CF_3_-PEA)_2_PbI_2_Br_2_ prepared by CF_3_-PEABr and PbI_2_.


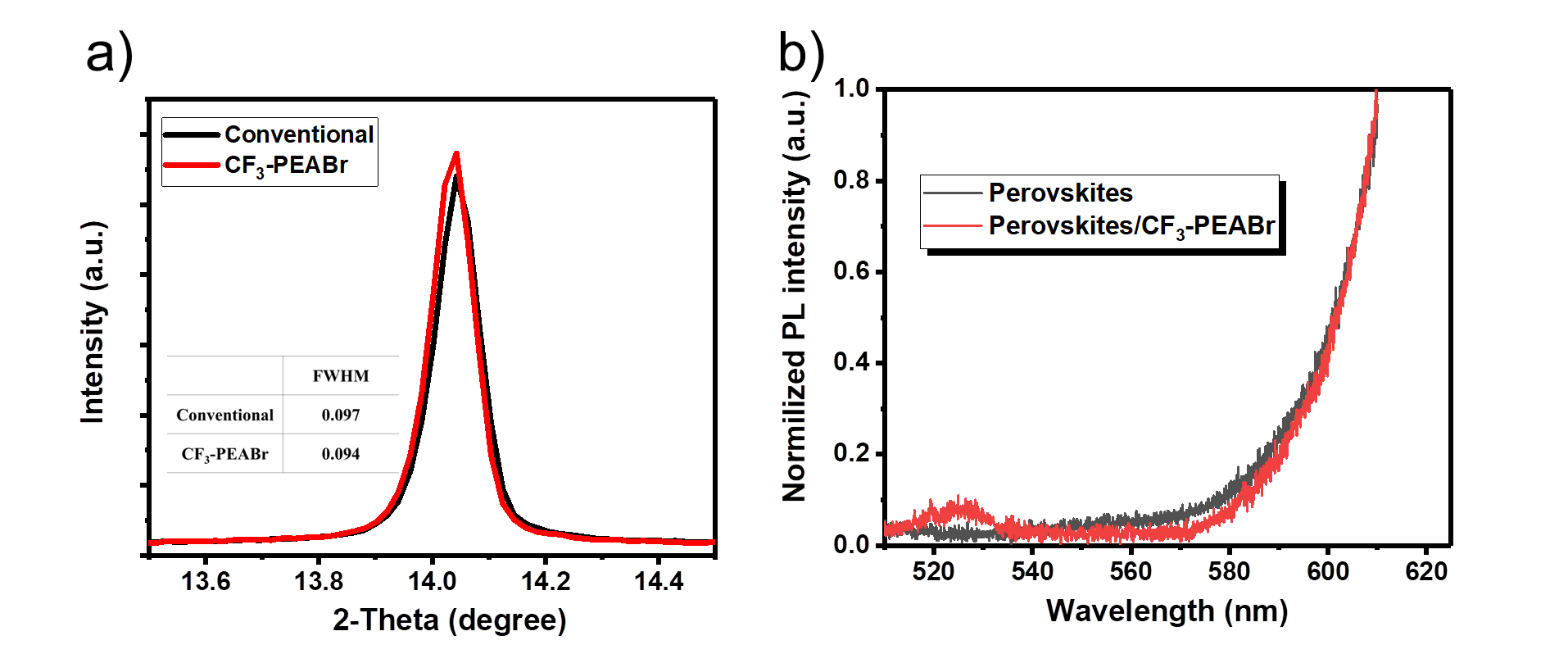


**Figure S2**. (a) FWHM of (110) peak based on Figure 1a. (b) PL spectra of perovskite films with or without CF_3_-PEABr passivation.

**Figure S3**. Tauc plots of the perovskite films with or without CF_3_-PEABr passivation.

**Figure S4.** AFM images of perovskite films with or without CF_3_-PEABr passivation.

**Figure S5**. FTIR spectra of CF_3_-PEABr powder and perovskite films.

**Figure S6**. XPS spectra of the provskite films with or without CF_3_-PEABr passivation.

**Figure S7**. UPS results of perovskites and band energy level scheme of different layers.

**Figure S8**. KPFM images of perovskite films: (a) conventional perovskite film, and (b) the perovskite film with CF_3_-PEABr passivation.

**Figure S9**. The device performance for samples with or without Triton X-100.

**Figure S10.** The schematic chemical structure of Triton X-100.

**Figure S11**. The performance of device with PEABr surface passivation.

**Figure S12**. The device performance of PSCs with different concentrations of Triton X-100 (Perovskites/PCBM without CF_3_-PEABr is used).

**Figure S13**. The device performance of PSCs with different concentrations of CF_3_-PEABr (Perovskites/CF_3_-PEABr/PCBM(Triton X-100) is used).

**Figure S14**. Hysteresis behaviors of perovskites devices with different conditions.

**Figure S15**. EQE curves of perovskite devices with different conditions.


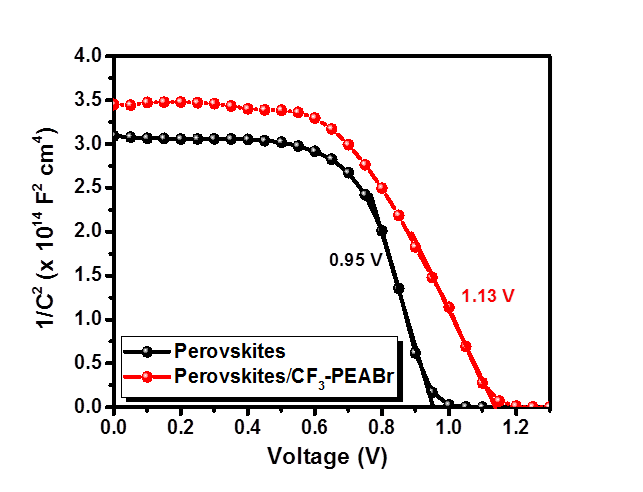


**Figure S16.** *C* – *V* curves of devices with or without CF_3_-PEABr treatment.

**Figure S17.** The illumination stability of films with or without CF_3_-PEABr treatment.

**Figure S18**. Cross-sectional SEM images of perovskite films: a) ITO/perovskites/PCBM, and b) ITO/perovskites(CF_3_-PEABr)/PCBM(Triton X-100)

**Table S1**. Fitting decay times of perovskite films prepared with different conditions. τ_1_ and τ_2_ are the lifetimes of fast decay and slow decay, respectively. A_1_ and A_2_ are the fractions of the fast decay and slow decay part, respectively.

|  | A_1_ | *τ_1_* (ns) | A_2_ | *τ_2_* (ns) | *τ*_ave_ (ns) |
| --- | --- | --- | --- | --- | --- |
| Perovskite | 0.21 | 28.4 | 0.79 | 132.5 | 127.4 |
| Perovskite/CF_3_-PEABr | 0.17 | 19.1 | 0.83 | 272.5 | 269.1 |

**Table S2**. Fitting decay times of perovskite films prepared with different conditions. τ_1_ and τ_2_ are the lifetimes of fast decay and slow decay, respectively. A_1_ and A_2_ are the fractions of the fast decay and slow decay part, respectively.

|  | A_1_ | *τ_1_* (ns) | A_2_ | *τ_2_* (ns) | *τ*_ave_ (ns) |
| --- | --- | --- | --- | --- | --- |
| Perovskite/PCBM | 0.38 | 2.19 | 0.62 | 8.12 | 7.24 |
| Perovskite/PCBM (Triton X-100) | 0.41 | 1.51 | 0.59 | 4.90 | 4.30 |

**Table S3.** Fitting parameters of the EIS measurments with different conditions.

|  | R_s_ (Ω) | R_rec_ (kΩ) |
| --- | --- | --- |
| Perovskite | 23.6 | 20.5 |
| Perovskite/CF_3_-PEABr | 26.7 | 33.1 |

Equivalent circuit:


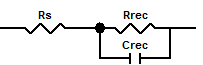


**Reference:**

1. Zhou, L., Lin, Z., Ning, Z., Li, T., Guo, X., Ma, J., Su, J., Zhang, C., Zhang, J., Liu, S., *et al.* (2019). Highly Efficient and Stable Planar Perovskite Solar Cells with Modulated Diffusion Passivation Toward High Power Conversion Efficiency and Ultrahigh Fill Factor. Sol. RRL *3*, 1900293.

2. Hedin, L., and Lundqvist, B.I. (1971). Explicit local exchange-correlation potentials. J. Phys. C Solid State Phys. *4*, 2064–2083.

3. Kresse, G., and Furthmüller, J. (1996). Efficiency of ab-initio total energy calculations for metals and semiconductors using a plane-wave basis set. Comput. Mater. Sci. *6*, 15–50.

4. Kresse, G., and Furthmüller, J. (1996). Efficient iterative schemes for ab initio total-energy calculations using a plane-wave basis set. Phys. Rev. B *54*, 11169–11186.

5. Kohn, W., and Sham, L.J. (1965). Self-Consistent Equations Including Exchange and Correlation Effects. Phys. Rev. *140*, A1133–A1138.

6. Kresse, G., and Joubert, D. (1999). From ultrasoft pseudopotentials to the projector augmented-wave method. Phys. Rev. B *59*, 1758–1775.

7. Blöchl, P.E. (1994). Projector augmented-wave method. Phys. Rev. B *50*, 17953–17979.

8. Perdew, J.P., Burke, K., and Ernzerhof, M. (1996). Generalized Gradient Approximation Made Simple. Phys. Rev. Lett. *77*, 3865–3868.

9. Henkelman, G., Uberuaga, B.P., and Jónsson, H. (2000). Climbing image nudged elastic band method for finding saddle points and minimum energy paths. J. Chem. Phys. *113*, 9901–9904.

10. Henkelman, G., and Jónsson, H. (2000). Improved tangent estimate in the nudged elastic band method for finding minimum energy paths and saddle points. J. Chem. Phys. *113*, 9978–9985.
